# Jingmen tick virus: an emerging arbovirus with a global threat

**DOI:** 10.1128/msphere.00281-23

**Published:** 2023-09-13

**Authors:** Zhen Wu, Ming Zhang, Yuli Zhang, Ke Lu, Wenbing Zhu, Shuo Feng, Jun Qi, Guoyu Niu

**Affiliations:** 1 WeiFang Medical University, Weifang, Shandong, China; 2 Tianjin Customs Port Out-Patient Department, Tianjin International Travel Healthcare Center, Tianjin, Hebei, China; University of Michigan, Ann Arbor, Michigan, USA

**Keywords:** Jingmen tick virus, tick, arboviruses, *Flaviviridae*, epidemiology, molecular biology

## Abstract

Jingmen tick virus (JMTV), belonging to the *Flaviviridae* family, is a novel segmented RNA virus identified in 2014 in the Jingmen region of Hubei Province, China. Up to now, JMTV has been detected in a variety of countries or regions in Asia, Europe, Africa, and the Americas, involving a wide range of arthropods and mammals, and even humans. The JMTV genome is composed of four linear RNA segments, two of which are derived from flaviviruses, while the other two segments are unique to JMTV and has no matching virus. Currently, JMTV has been shown to have a pathogenic effect on humans. Humans who had been infected would develop viremia and variable degrees of clinical symptoms. However, the pathogenic mechanism of JMTV has not been elucidated yet. Therefore, it is crucial to strengthen the epidemiological surveillance and laboratory studies of JMTV.

## INTRODUCTION

Jingmen tick virus (JMTV), a novel tick-borne RNA virus, was first identified in 2014 in *Rhipicephalus microplus* from the Jingmen region of Hubei Province, China (1). JMTV is considered as unclassified *Flaviviridae* by the International Committee on Classification of Viruses but differs from typical flaviviruses in that the JMTV genome has four segments, of which segment 1 and segment 3 are associated with non-structural protein (NSP) genes of the genus *Flavivirus*, whereas the remaining two segments are specific to JMTV (1, 2). This segmented genomic signature of JMTV has led to changes in the public understanding of flavivirus genome structure while revealing unexpected evolutionary links between non-segmented and segmented RNA virus genomes (3). The recent researches indicated that JMTV has been detected in a variety of arthropods, such as ticks, *Culex pipiens*, and *Drosophila melanogaster*, suggesting a wide host spectrum for JMTV (4 – 6). In addition, antibodies or genomes of JMTV have been successively detected in mammals such as cattle and bats, as well as in many rodents. More importantly, JMTV RNA was detected in fatal cases of Crimean-Congo hemorrhagic fever virus (CCHFV) in Kosovo and in tick-bite patients from China (7). In the meanwhile, JMTV was shown to be present and replicate in human skin tissue by *in situ* hybridization (7, 8). These results indicated JMTV has a potential pathogenicity to humans.

JMTV has been reported in many regions of northeastern, northwestern, central, and southern China, as well as in many countries in continental Europe and the Americas since JMTV was first described in Hubei Province, China (9 – 11). In recent years, many viruses with similar genome structure to JMTV have also been identified, such as Alongshan virus (12), Mogiana tick virus (13), and Guaico culex virus (2), forming the Jingmen virus group. Up to now, the data on the Jingmen virus group including JMTV were mostly from the results of metagenomics, and its transmission cycle, infectivity, and pathogenesis still remained to be further elucidated. This review focused on the global distribution, hosts, genomic features, and phylogenetic relationships of JMTV, providing a theoretical baseline for further research on JMTV.

## EPIDEMIOLOGY

### Geographic distribution

**Fig 1:**
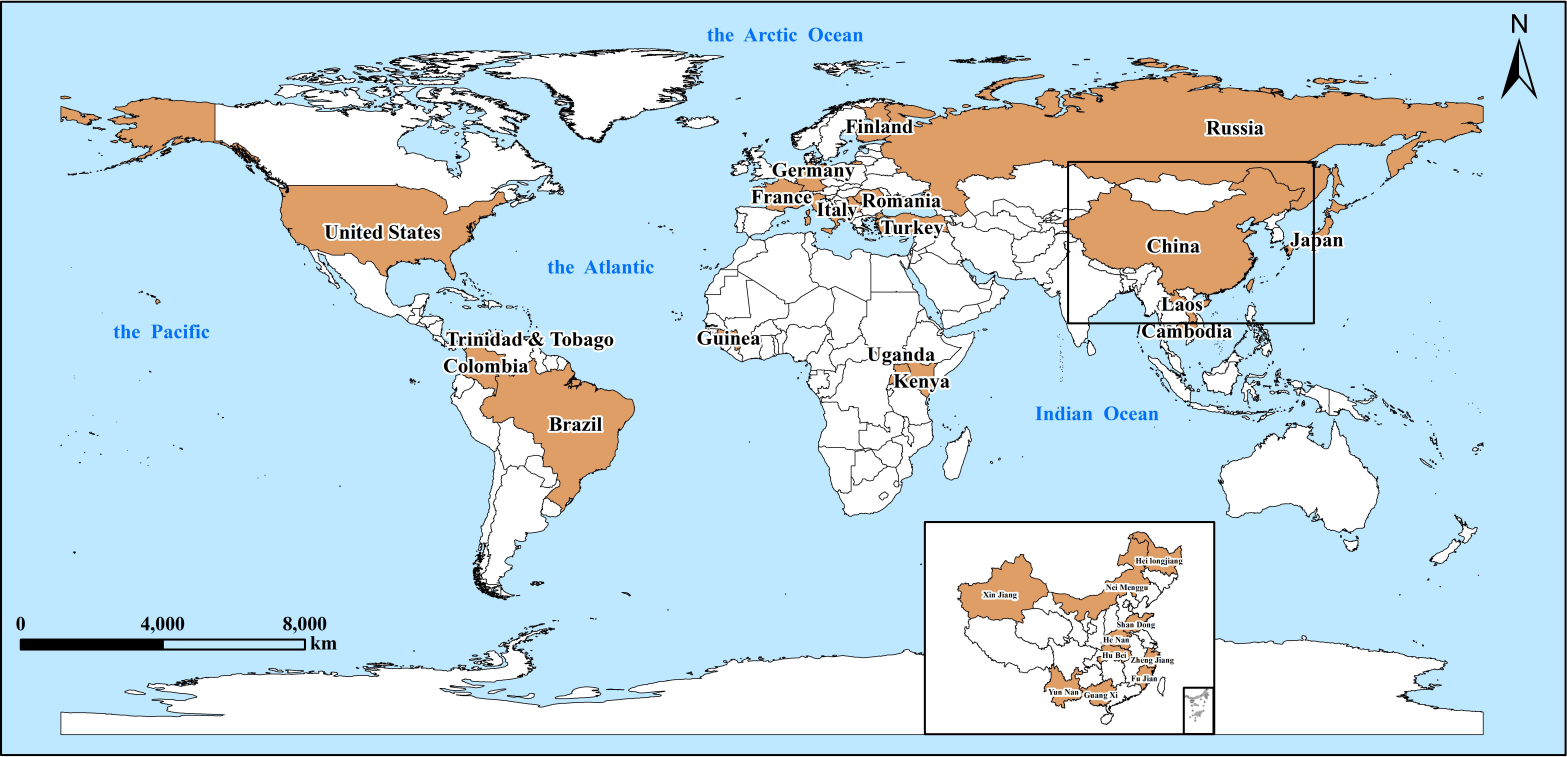
The global distribution of JMTV from 2014 when it was first reported to date. The areas highlighted in orange color indicate the countries or regions where JMTV was identified.

JMTV had an extremely wide geographical distribution, covering a number of countries and regions in Asia, Europe, Africa, and the Americas. The earliest detailed description of JMTV appeared in 2014 when it was detected in the Jingmen region of Hubei Province, China. Since then, JMTV has been reported to be endemic and spreading in many areas of China, including Zhejiang, Yunnan, Fujian, Xinjiang, Guangxi, and Heilongjiang provinces (7, 14 – 16). In the meanwhile, JMTV has also been identified outside of China, for example, in Brazil and Uganda in 2016, Kosovo in 2018, Lao PDR, Turkey, Kenya, France, and North America in 2019, Colombia and Japan in 2020, and Italy in 2021 (5, 11, 17 – 20) (Table 1). With the increased focus on arboviruses and the continued development of next-generation sequencing technologies (16), an increasing number of arbovirus screening and genomic studies were being conducted, and JMTV was detected in more extensive regions. To date, JMTV genome sequences have been detected in samples from four continents worldwide (Fig. 1).

**TABLE 1:** Characteristics of JMTV genome from different countries and regions

Virus	Host	Strain	Location	Protein	Isolation	Reference
JTMV	*R. microplus*, *H. longicornis*, *H. campanulata*, *I. granulatus*, *H. flava*, *I. sinensis*, *Bos* sp., *R. sanguineus*, *Armigeres* sp.	SY84	China	NSP1:914aa, VP1:754aa; NSP2:808aa, VP2:254aa, VP3:538aa	Yes	(1)
	*Amblyomma javanense*	GXTV108			Yes	(7)
	*Microtus arvalis*, *Apodemus uralensis*, *Rhombomys opimus*, *Mus musculus*, *Meriones tamariscinus*, *Meriones libycus*, *Microtus gregalis*, *Cricetulus migratorius*	XJ58			–^*a*^	(3)
	*Haemaphysalis hystricis*, *R. microplus*, *Bubalus bubalis*, *Rattus tanezumi*, *Rattus norvegicus*, *Apoodemus agragius*, *Myotis davidii*, *Myotis laniger*, *Miniopterus fuliginosus*, *Nyctalus noctula*, *Pipistrellus abramus*, *Eptesicus serotinus andersoni*, *Rhinolophus sinicus*, *Myotis siligorensis*, *Rhinolophus pusillus*, *Rhinolophus pearsonii*, *Rhinolophus ferrumequinum*	WZHu11; WZRm10; WZBb28; WZRt4; WZMd125; ALMd316; ALMl377; WZMf170; ALRs134; ALMs423; NXRpu28; NXRpe39			–	(3)
	*R. microplus*; *Hae. longicornis*	HAHHY1; LTBMH8; HAHY1; SZYD2; SZWH1			–	(21)
	*R. microplus*	YNflaviV			No	(16)
	*H. longicornis*	ZYJ2; ZYJ4			No	(4)
	Pregnant sow	–			–	(14)
	*I. persulcatus*	–			–	(22)
	*A. testudinarium*, *H. longicornis*	WS3			No	(15)
	*Homo sapiens*	–			–	(8)
	*R. microplus*	JTMV_1; JTMV_3; JTMV_100	Brazil	NSP1:914aa, VP1:753aa; NSP2:808aa, VP2:254aa, VP3:501aa	No	(17)
	*Homo sapiens*	Kosovo 2013-17-266; Kosovo 2014-C-K14-1C; Kosovo 2015-A-K15-1A	Kosovo	NSP1:914aa, VP1:744aa; NSP2:808aa, VP2:254aa, VP3:538aa	–	(23)
	*R. bursa*, *Rhipicephalus turanicus*	T14; T17; T36	Turkey	NSP1:914aa, VP1:744aa; NSP2:808aa, VP2:254aa, VP3:538aa	–	(24)
	*Ixodes ricinus*	JMTV/*I. ricinus*/France	France	NSP1:914aa, glycoprotein 1: 481aa, glycoprotein 2: 266aa; NSP2:810aa, VP2:252aa, VP3:484aa	–	(11)
	*R. microplus*	JMTV/*Rh. microplus*		NSP1:914aa, VP1:744aa; NSP2:808aa, VP2:254aa, VP3:538aa		
	*A. testudinarium*	JMTV/*Am. testudinarium*	Laos	NSP1:914aa, VP1:754aa; NSP2:808a, VP2:254aa, VP3:538aa		
	*Pteropus lylei*	JMTV/*Pteropus lylei*	Cambodia	NSP1:914aa, VP1:735aa; NSP2:666a, VP2:265aa, VP3:471aa		
	*R. microplus*	TTP-Pool-3b	Trinidad and Tobago	NSP1:914aa, VP1:744aa; NSP2:777aa, VP2:254aa, VP3:501aa	–	(25)
	Histiostoma	018	Germany	NSP1:1034aa, VP1:734aa; NSP2:947aa, VP2:256aa, VP3:574aa	–	(6)
	*Aedes albopictus*	RIMINI	Italy	NSP1:905aa, VP1:744aa; NSP2:808aa, VP2:254aa, VP3:538aa	–	(26)
	*A. testudinarium*	18EH12,32; 19EH-IM24, IM-OI12,96,108,119; ISK55	Japan	NSP1:914aa, VP1:754aa; NSP2:808a, VP2:254aa, VP3:538aa	No	(20)
	*Rhipicephalus bursa*	Tulcea1	Romania	NSP1:914aa, VP1:744aa; NSP2:808a, VP2:254aa, VP3:501aa	–	(27)
	*Am.* sp., *Am. sparsum*, *Rhipicephalus appendiculatus*, *Rhipicephalus evertsi evertsi*, *Hyalomma truncatum*, *Amblyomma nuttalli*, tortoise	MT297,299,304,308,328; MT29,293	Kenya	NSP1:914aa, VP1:754aa; NSP2:808a, VP2:254aa, VP3:538aa	No	(28)

^*a*^ “–”: the relevant content was not mentioned in the literature.

### Transmission and vector control

The primary mode of transmission of JMTV was likely to occur through tick bites during blood-sucking. When infected ticks bit hosts, JMTV that accumulated in the salivary glands was transmitted to the recipient via the oral organs. In addition, JMTV could be transmitted transovarially to offspring by female ticks. Currently, JMTV has been shown to be transmitted vertically to offspring by *Haemaphysalis longicornis* and *Amblyomma testudinarium* via the transovarial route (9, 29). Meanwhile, JMTV may be transmitted between ticks through a non-viremic host by co-feeding. Briefly, JMTV underwent tick-host-tick transmission when ticks fed on a non-viremic or virus-immune host (26, 30). Ticks, the second most pathogenic vector of infection after mosquitoes, were the primary hosts for carrying and transmitting JMTV. JMTV was originally found in *Rhipicephalus microplus*. Subsequently, JMTV was detected in a variety of ticks including *Haemaphysalis longicornis*, *Haemaphysalis campanulata*, *Haemaphysalis flava*, *Rhipicephalus sanguineus*, *Ixodes sinensis*, *Ixodes granulatus*, and *Amblyomma javanense* (Table 1). Despite regional variations in the species of ticks reported to have detected JMTV, to date, the most common species for which JMTV RNA has been detected is *Rhipicephalus microplus*. Data from recent studies showed that high prevalence of JMTV has been found in *Rhipicephalus microplus* from China (53%–63%), Brazil (25%–67%), Trinidad and Tobago (6%–46%), and the French Antilles (24%–77%) (9). Nevertheless, JMTV RNA was also detected in *Drosophila melanogaster* and two species of mosquitoes including *Armigeres* spp. and *Anopheles* spp. These results suggested that the virus may use multiple arthropods as vectors for transmission. In addition to these arthropods, JMTV genome has been detected in many mammals. For example, JMTV has been identified in cattle in Brazil, in bats in Anatolia, in goats in Kenya, and in rodents in China and Pennsylvania, North America (3, 7, 28). These results indicated that JMTV had an extremely broad host spectrum and was potentially pathogenic to animals, but the symptoms caused by JMTV ranging from mild to severe need to be further elucidated. More importantly, the genome of JMTV was detected in patients with CCHFV patients in Kosovo and in febrile patients in China (12, 23). These results suggested JMTV is pathogenic to humans and may be co-infected with other viruses.

## GENOME ANALYSIS

**Fig 2:**
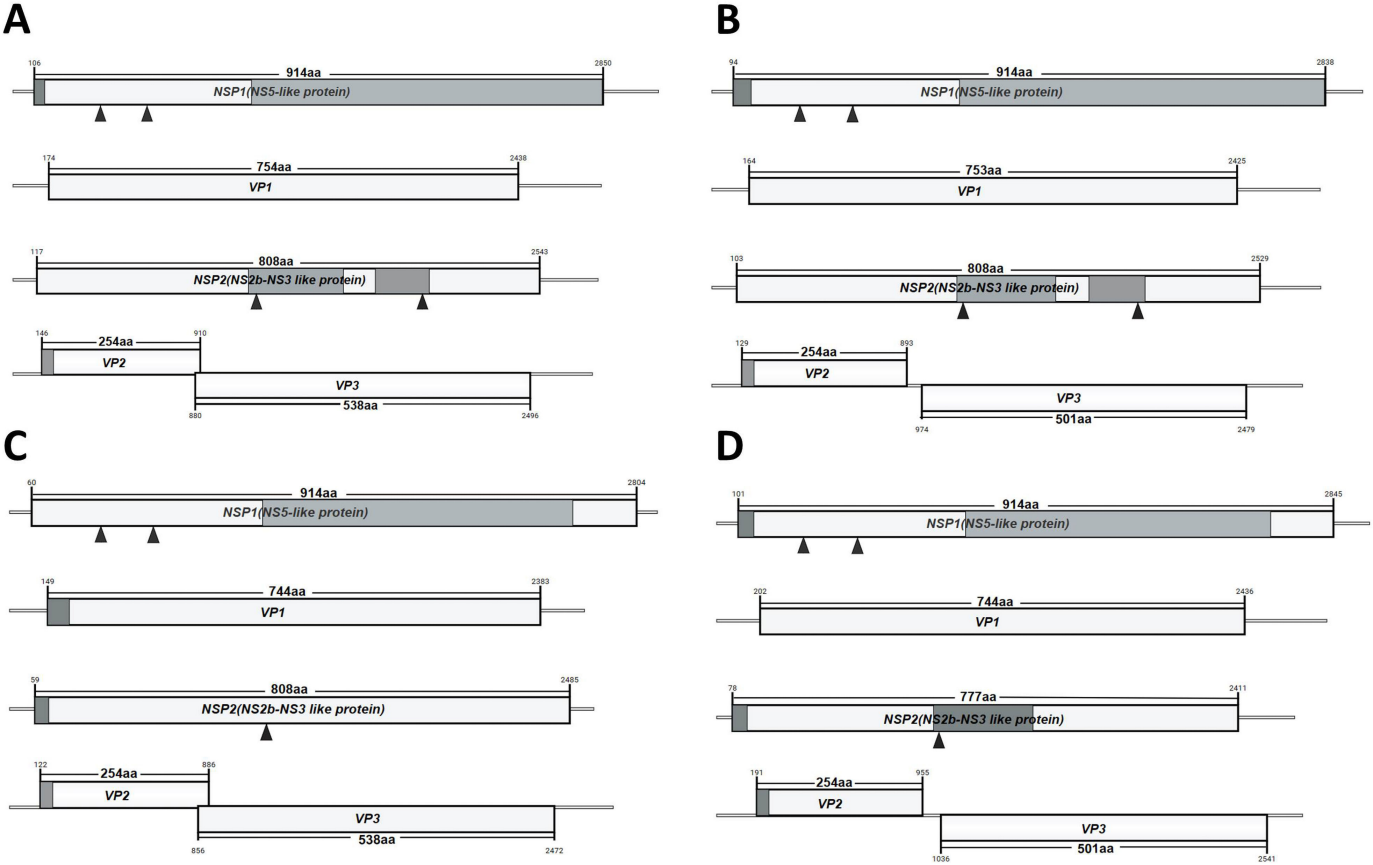
Structural features of four segments of the genomic sequence of JMTV. The four genomic sequences of JMTV were used to analyze and compare the genomic structure of JMTV. (A) JMTV strain from China. (B) JMTV strain from Brazil. (C) JMTV strain from Turkey. (D) JMTV strain from Trinidad and Tobago.

Unlike typical flaviviruses, the JMTV genome is composed of four segments, S1, S2, S3, and S4. Segments 1, 2, and 3 are monocistronic with an open reading frame (ORF) that encodes a protein, while segment 4 is bicistronic, containing two ORFs and encoding two proteins. Fluorescence *in situ* hybridization results suggested that the four segments of JMTV might be packaged in a single viral particle (7). This segmented evolution usually demonstrates a high rate of virus evolution (1), making it possible to efficiently express all groups of viral genes and contributing to the stability of the virus. Segment 1 and segment 3 gene sequences encode the NSPs of JMTV, NSP1, and NSP2 (Fig. 2). Protein sequence alignment analysis showed that NSP1 and NSP2 of JMTV have high similarity to the non-structural proteins NSP5 and NSb2-NS3 of flavivirus, and it is inferred that the sequences of JMTV segment 1 and segment 3 may be derived from flavivirus. In addition, segment 1 has a conserved motif for the flavivirus RNA-directed RNA polymerase, as well as an S-adenosylmethionine binding site and a nucleic acid substrate binding site. The N-terminus of NSP1 encoded by JMTV segment 1 also has a 27-amino acid signal peptide to facilitate protein synthesis, which is why NSP1 was slightly longer than NS5 (1). In the meanwhile, segment 3 also has two conserved motifs, N-terminal helicase domain and C-terminal helicase domain, together with an ATP binding site. Interestingly, the proteins encoded by segment 3 of the French, Cambodian, and Trinidad and Tobago JMTVs were significantly different from those encoded by JMTV strains from other sources. The NSP2 encoded by segment 3 of Cambodian and Trinidad and Tobago JMTV had only 666 aa and 777 aa, respectively, which were shorter than those encoded by the other JMTVs. Whereas, the NSP2 encoded by segment 3 of the French and German JMTVs had 810 aa and 947 aa, respectively, which were longer than the NSP2 encoded by the other JMTVs (Table 1). This may be attributed to genetic mutations that have occurred during the transmission and evolution of JMTV. Segment 2 and segment 4 of JMTV are responsible for encoding the structural proteins of JMTV (Fig. 2). Currently, no viral sequences matching segment 2 and segment 4 have been found; thus, they may have originated from as yet undiscovered viruses. Segment 2 has an ORF responsible for encoding JMTV glycoprotein VP1, while segment 4 has two overlapping ORFs for the encoding of capsid protein VP2 and membrane protein VP3, respectively. Furthermore, there is a 19-amino acid signal peptide at the N-terminal of the capsid protein (VP2) encoded by segment 4. In addition, the structural proteins encoded by the two RNA segments (segment 2 and segment 4) that are unique to JMTV exhibit different sizes depending on the virus strain. Segment 2 of most JMTV strains from European countries encodes the glycoprotein VP1 which is 744 amino acids, segment 2 of JMTV from Brazil and Guinea encodes 753 amino acids, and the glycoprotein of JMTV from China and other regions is 754 amino acids. Importantly, segment 2 of a strain of JMTV from France has two open ORFs encoding glycoprotein 1 and glycoprotein 2, which is due to an ORF disruption caused by a mutation in the gene, and the resulting changes in the proteins need to be further investigated. Meanwhile, JMTV from Uganda encodes a glycoprotein (VP1) of only 604 aa, and sequence analysis showed that there was a base loss in this sequence (Table 1). Segment 4 encodes a membrane protein (VP3) similar to VP1, consisting of 505 amino acids for JMTV glycoproteins from Brazil and Trinidad and Tobago, and 538 amino acids in other JMTV glycoproteins. Meanwhile, JMTV from countries such as China, Turkey, and Uganda has two ORFs overlapping in segment 4, but JMTV segment 4 from Brazil and Trinidad and Tobago has only one ORF. These results indicated that JMTV underwent macroevolution or genetic mutation during adaptation to the local environment and that these changes enabled the virus to overcome the pressures of natural selection (20, 29).

## PHYLOGENETIC ANALYSIS

Pairwise distance analysis of all available JMTV genome sequences showed that, with the exception of the JMTV 018 strain from Germany, the nucleic acid identity of segment 1, segment 2, segment 3, and segment 4 of all JMTV strains was 69.4%–100%, 57.4%–100%, 68%–99.9%, and 59.5%–100%, respectively. There were significant differences between JMTV strain 018 from Germany and the currently published sequences of other JMTV strains. Specifically, segments 1, 2, 3, and 4 of JMTV strain 018 shared only 46%–48.2%, 32.1%–38%, 41.1%–42.7%, and 38%–40.3% nucleotide identity with the sequences of other JMTVs, respectively (Tables S1 to S5). JMTV strain 018 was isolated from Histiostoma, and the biology of the host and the intervention of its immune system may lead to mutations or even rearrangements in the viral genes, resulting in large differences in the JMTV sequence (31).

**Fig 3:**
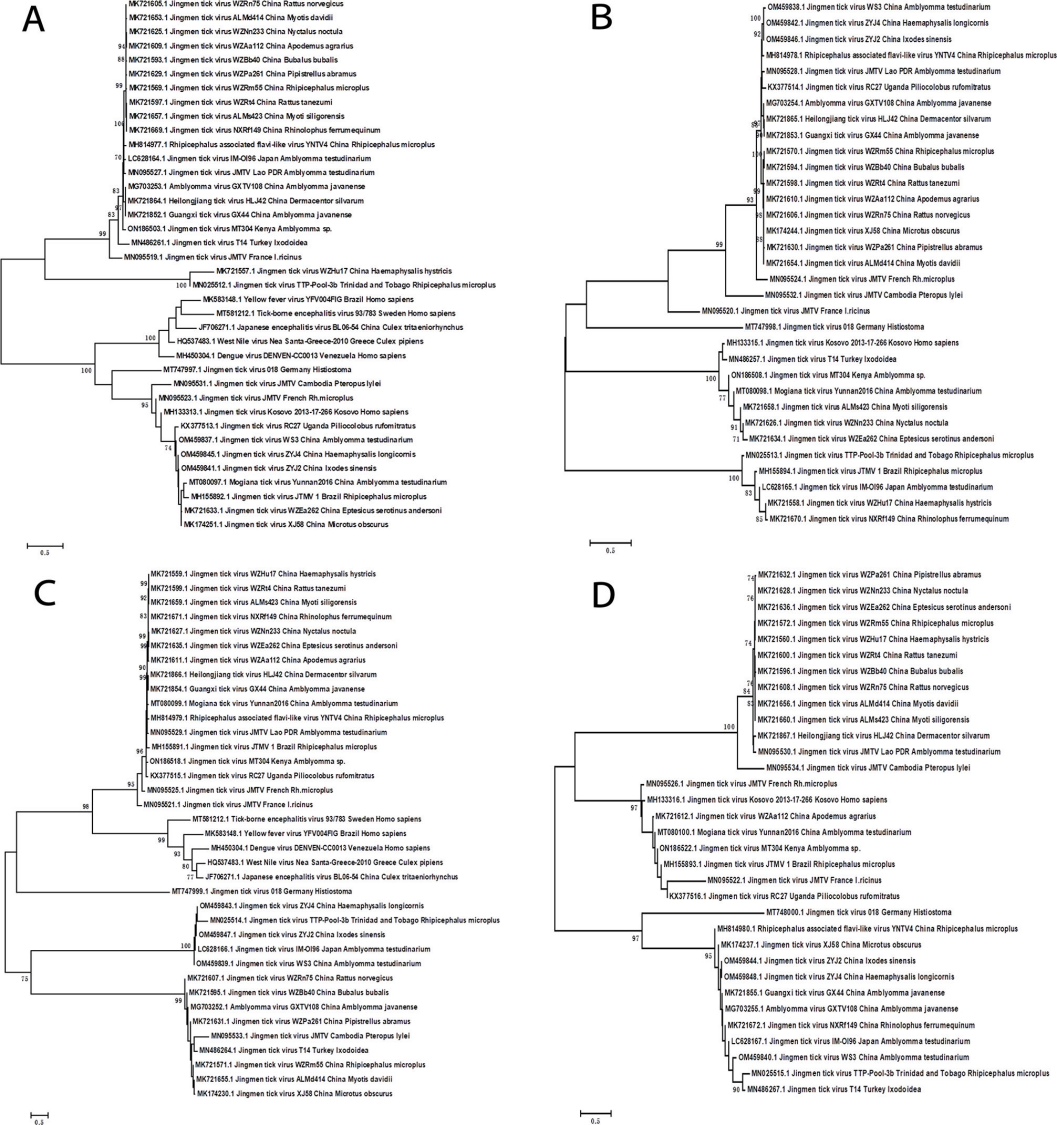
Phylogenetic analysis of the complete sequences of JMTV strains. Phylogenetic trees were constructed by the neighbor-joining (NJ) method using MEGA 5.1, with 1,000 replicates with bootstrap values >70% considered significant. The numbers above the branches indicate bootstrap values. Phylogenetic analysis of JMTV using the whole nucleotide sequences of (A) segment 1, (B) segment 2, (C) segment 3, and (D) segment 4, respectively.

The phylogenetic tree was constructed by neighbor-joining method to better analyze the phylogenetic relationships of JMTV, and the reliability of the tree was determined by the bootstrap method (1,000 repetitions). The bootstrap values >70% are considered to be significant. All available JMTV and JMTV-related virus sequences could be divided into two major phylogenetic groups, with the first group containing JMTV identified from China, Japan, France, Turkey, Kenya, Tobago together with Amblyomma virus, Rhipicephalus-associated flavi-like virus, Heilongjiang tick virus, and Guangxi tick virus from China, while the second group was composed of JMTV and typical flaviviruses from Asia, Europe, Africa, and South America (Fig. 3). Within the second group, all sequences could be further divided into two subgroups. Subgroup I comprised Japanese encephalitis virus from Asia, Tick-borne encephalitis virus and West Nile virus identified in Europe, as well as Dengue and Yellow fever viruses isolated from the Americas. All JMTV strains from Germany, Cambodia, French, Kosovo, Uganda, China, and Brazil clustered with Mogiana tick virus from China formed subgroup II. The composition of the first group clearly revealed that JMTV identified from different species in China was closely clustered together and highly homologous. These results were consistent with the vectorial transmission cycle, suggesting that species isolation would not affect the genetic diversity of JMTV, and JMTV was strongly adaptable to hosts (3). In addition, the spatial segregation of viral subclades was observed. This result was consistent with the results of the identity analysis based on the whole genome and amino acids of JMTV, showing that the gene segments and the amino acids encoded by the JMTV 018 strain were significantly different from those of other JMTV strains (Tables S1 to S5). This indicated that there may be separate lineages of JMTV strains or that JMTV had evolved in different directions in different geographical locations and hosts.

## VIRUS ISOLATION

A variety of arthropod and mammalian cells including Vero, Vero E6, BHK-21, C6/36, BME26, DH82, and BME/CTVM23 have been used for viral isolation of JMTV (1, 16, 17). Despite live viruses were successfully isolated from JMTV-infected Vero, Vero E6, C6/36, and DH82 cells, the virus was not detectable in other cell lines after two passages. Even so, the isolation of these cells from JMTV was not always successful. At present, JMTV was only stably transmitted in the BME/CTVM23 cell line (7). The reason for this may be associated with differences in the growth cycle of every cell line. Tick-derived BME/CTVM19 cells have a cell replication cycle of 10 days, whereas C6/36, DH82, and Vero cells have a passaging cycle of approximately 0.9–2 days (20, 32). These results also implied that different cell lines may have an important influence on the replication of JMTV, and BME/CTVM23 cells may be more suitable for the growth of JMTV. These results also implicated that different cell lines may have important effects on JMTV replication and that the *Ixodidae*-derived BME/CTVM23 cell line may be more suitable for the growth of JMTV isolated from *Ixodidae* (33). Finally, the possibility that the JMTV in these cells was a viral residue in the tick homogenate could not be excluded (28). DH82 and C3/36 cells, as an initial attempt to isolate JMTV, were successfully isolated to the JMTV strain, and cell detachment occurred in both cells during the process of JMTV virus infection. Unfortunately, no apparent cytopathic effect (CPE) was observed during JMTV replication, and the virus was undetectable following two passages in these DH82 and C3/36 cells (1, 24). Similar to DH82 and C6/36 cells, Vero and Vero E6 cells supported JMTV replication, and the JMTV genome was no longer detected after two passages. However, Vero E6 cells were observed to have CPE during viral infection, showing cytosolic elongation and vacuolization but no obvious vacuole formation (24). Transmission electron microscopy was performed to visualize DH82 cells infected by JMTV. There were numerous virus-like particles observed in the field of view. These virus-like particles are round, with envelopes and obvious protrusions, approximately 70–80 nm in diameter, which is slightly larger than normal flavivirus particles. The similar structures were not observed in JMTV-uninfected DH82. Therefore, these virus-like particles are most likely JMTV, and further studies are necessary to verify the virus particle morphology of JMTV (1, 9).

## PATHOGENICITY

JMTV is a novel tick-borne virus, for which studies on human and animal pathogenicity are extremely important. In 2014, an immunofluorescence assay was performed on 145 cattle sera from Hubei and Zhejiang provinces, 54 samples were positive for JMTV, indicating the presence of antibodies against JMTV in cattle blood (1). In 2016, a variant of JMTV was detected in the primate red colobus monkey in Uganda (5). Subsequently, in 2018, the JMTV genome was detected in serum samples from patients with Crimean-Congo hemorrhagic fever in Kosovo (23). These results suggested that JMTV was potentially pathogenic to humans and animals, and further research was required to better understand this novel virus. More importantly, replication of the virus has been detected in human skin tissue bitten by JMTV-infected ticks. The bitten skin showed itching, necrosis, inflammatory cell infiltration, and finally scorched crust formation (7). In addition, a retrospective serological test involving 509 patients revealed that JMTV antibodies were present in the serum of eight patients. These results provided the first direct evidence that JMTV was pathogenic to humans. Subsequently, JMTV antibodies were also detected sporadically in 70 patients with a history of tick bites in France (11). In brief, JMTV infection has been shown to cause viremia in humans and some mammals, including monkeys and cattle, and may cause a range of clinical symptoms from mild to severe in humans. However, there remains a lack of systematic studies on the pathogenic mechanisms of JMTV. Therefore, it has great significance to strengthen the research related to the epidemiology and pathogenesis of JMTV to better understand this novel virus and to deal with related diseases more effectively.

### Conclusion

JMTV is the first segmented RNA virus to be identified and confirmed, and is closely associated with the typical unsegmented flavivirus. A global survey on the epidemiology and pathogenic effects of JMTV has been conducted since its discovery in Hubei Province. It was reported that RNA of JMTV was found in four continental regions worldwide and involved multiple hosts including humans. Despite the fact that no human or animal mortality has been reported from JMTV, it has been shown that JMTV may cause viremia in humans or animals and that infected individuals may show variable degrees of clinical symptoms. More intensive laboratory studies and long-term epidemiological surveillance to elucidate the pathogenicity of JMTV in humans and animals and the global distribution of JMTV would be necessary to avoid possible local or large-scale epidemics caused by the virus, especially in regions where JMTV and JMTV-associated viruses have been detected.

In conclusion, we reviewed the recent advances of JMTV, outlined the geographic distribution and transmission vectors of JMTV, and analyzed the JMTV genome and evolutionary relationships to improve our understanding of the virus and develop effective control measures.

## SUPPLEMENTAL MATERIAL

Tables S1 to S5 msphere.00281-23-s0001.docx Characteristics of JMTV genome from different countries and regions.

ASM does not own the copyrights to Supplemental Material that may be linked to, or accessed through, an article. The authors have granted ASM a non-exclusive, world-wide license to publish the Supplemental Material files. Please contact the corresponding author directly for reuse.
