# Supplementary material for: Jingmen tick virus: an emerging arbovirus with a global threat
Source: mSphere. 2023 Sep 13;8(5):e00281-23. doi: 10.1128/msphere.00281-23 (PMC10597410; doi:10.1128/msphere.00281-23)
Supplement: Tables S1 to S5 — Characteristics of JMTV genome from different countries and regions. [file msphere.00281-23-s0001.docx]

**Supplementary Table 1. The nucleotide (bottom diagonal) and amino acid (top diagonal) sequence identities of segment 1 among the available JMTVs**

**Supplementary Table 2. The nucleotide (bottom diagonal) and amino acid (top diagonal) sequence identities of segment 2 among the available JMTVs**

**Supplementary Table 3. The nucleotide (bottom diagonal) and amino acid (top diagonal) sequence identities of segment 3 among the available JMTVs**

**Supplementary Table 4. The nucleotide (bottom diagonal) and amino acid (VP2) (top diagonal) sequence identities of segment 4 among the available JMTVs**

**Supplementary Table 5. The nucleotide (bottom diagonal) and amino acid(VP3) (top diagonal) sequence identities of segment 4 among the available JMTVs**
